# Prevalence of gonococcal and chlamydial infections among men who have sex with men in sub-Saharan Africa: protocol for a systematic review and meta-analysis

**DOI:** 10.1186/s13643-023-02305-2

**Published:** 2023-08-14

**Authors:** Kehinde Charles Mofolorunsho, Vinogrin Dorsamy, Chauntelle Bagwandeen, Nathlee Samantha Abbai

**Affiliations:** 1 School of Clinical Medicine Laboratory, College of Health Sciences, University of KwaZulu-Natal, KwaZulu-Natal, South Africa; 2 School of Laboratory Medicine and Medical Sciences, College of Health Sciences, University of KwaZulu-Natal, KwaZulu-Natal, South Africa; 3 School of Nursing and Public Health, College of Health Sciences, University of KwaZulu-Natal, KwaZulu-Natal, South Africa

**Keywords:** Prevalence, Men who have sex with men, *Neisseria gonorrhoeae*, *Chlamydia trachomatis*, Sub-Saharan Africa

## Abstract

**Background:**

Bacterial sexually transmitted infections (STIs) including *Neisseria gonorrhoeae* and *Chlamydia trachomatis* are common in men who have sex with men (MSM). These infections increase the risk of acquiring and transmitting human immunodeficiency virus (HIV) in this key population. Access to MSM in many countries in sub-Saharan Africa remains generally difficult due to discrimination or criminalization of their sexual orientation which could lead to depression and risky sexual practices associated with prevalence. This protocol therefore proposes to undertake a systematic review and meta-analysis of literature on the prevalence of gonococcal and chlamydial infections among MSM in Sub-Saharan Africa.

**Methods:**

This review which aims to ascertain the pooled prevalence and risk factors of these infections in sub-Saharan Africa’s MSM population will follow the Preferred Reporting Items for Systematic Review and Meta-analyses (PRISMA) guidelines. The search strategy will review relevant articles from the following databases: PubMed, Scopus, ISI Web of Science and the Directory of Open Access Journals (DOAJ). Articles screening for eligibility and data extraction will be conducted by two independent reviewers. All discrepancies will be resolved by the third and fourth reviewers. Heterogeneity in studies will be evaluated using the *I*^*2*^ statistic and where heterogeneity is high and significant, a random effect model will be used to estimate the pooled prevalence. Publication bias will be assessed using the Doi plot. Extracted data will be analysed using MetaXL add-on for Microsoft excel. Data will be presented in tables and graphically presented in forest plots.

**Discussion:**

In this study, we anticipate being able to systematically determine the prevalence of *Neisseria gonorrhoeae* and *Chlamydia trachomatis* among MSM as well as explore possible risk factors associated with prevalence. The outcomes of the systematic review and meta-analyses will serve to support researchers and public health stakeholders in identifying healthcare priorities and in addressing issues pertaining to the overall wellbeing of the MSM community.

**Systematic review registration:**

PROSPERO CRD42022327095

## Background

Sexually transmitted infections (STIs), including gonorrhoea and chlamydia, cause significant morbidity and mortality worldwide [1]. These STIs are a variety of clinical syndromes caused by pathogens that can easily be transmitted through sexual contact [2] and can facilitate individual susceptibility to human immunodeficiency virus (HIV) acquisition and transmission [1, 3]. Globally, the rate of STIs is increasing despite biomedical advances and considerable public health efforts. In 2012, 498.9 million new cases of curable STIs were reported worldwide. Their burden is disproportionally higher in low- and middle-income countries, with sub-Saharan Africa contributing 93 million new cases [4, 5]. Vulnerable populations include men who have sex with men (MSM) [6].

Studies on MSM in sub-Saharan Africa have reported high prevalence of STIs including *Neisseria gonorrhoea* (Ng) and *Chlamydia trachomatis* (Ct) [7, 8]. These bacterial infections (Ng and Ct) among African MSM have prevalence reportedly ranging from 1% [9] to 23% [10]. Studies from Kenya have shown Ng prevalence among gay men and other MSM to be 9.3% [11]. In Senegal, Ng prevalence among MSM has been estimated at 5.5% [12, 13] while in Nigeria, a published study estimated a Ng prevalence of 23% and Ct prevalence of 16% [14].

Although much attention has been given to African MSM in recent times, access to MSM in many countries in sub-Saharan Africa remains generally difficult. This situation is largely due to stigma, discrimination and/or criminalization of their sexual orientation [15–17], leading to their reluctance in accessing healthcare services. Because MSM suffer significant stigma due to lack of social acceptance, they become depressed and may engage in risky sexual practices including alcohol and substance abuse which are associated with prevalence [18–20]. Almost two-third of African countries still criminalize same-sex sexual behaviour, with long prison sentences or even the death penalty [21]. These issues may explain why research on MSM in sub-Saharan Africa is lagging behind other parts of the world [22–24], contributing to the paucity of information on this key population at higher risk for STIs. Hence, a systematic review aimed at ascertaining the pooled prevalence of Ng and Ct, and their associated risk factors in MSM in sub-Saharan Africa is necessary. The result of which is likely to inform research and policies in identifying healthcare priorities for this population living in Africa.

### Objectives

This protocol for a systematic review aims to document the pooled prevalence of infections by *Chlamydia trachomatis* and *Neisseria gonorrhoeae* in MSM in sub-Saharan Africa. Other objectives are to:

- Describe chlamydial and gonococcal infections diagnosed by syndromic management or laboratory testing
- Identify the associated risk factors for the prevalent infections among MSM

## Methods

This protocol is registered with the open access registry for systematic review protocols PROSPERO (CRD42022327095) and developed in accordance with the Preferred Reporting Items for Systematic Review and Meta-analyses for Protocols (PRISMA-P) 2015 checklist [25]. The planned systematic review will be reported according to the PRISMA 2020 statement [26].

### Eligibility of research question

A Population Exposure Outcomes (PEO) framework (Table 1) will be used to evaluate the eligibility of the research question. Only studies reporting overall and/or anatomical site-specific prevalence of chlamydia and/or gonorrhoea in men who have sex with men will be included. No restriction will be applied for study design, diagnostic test type and year of publication.

**Table 1:** PEO (Population, Exposure, Outcomes) framework

Population	MSM in sub-Saharan Africa
Intervention/Exposure	Prevalence of chlamydia and gonorrhoea infections determined by syndromic management or laboratory testing for urethral/pharyngeal/rectal infection
Outcomes	Overall and anatomical site-specific prevalence rates, and risk factors for chlamydia and gonorrhoea

### Identifying relevant studies

Electronic databases will be used to search articles in peer-reviewed journals from the following databases: PubMed, Scopus, ISI Web of Science, Directory of Open Access Journals (DOAJ). Search terms used and their synonyms were identified using the Medical Subject Headings (MeSH). The uniterms and Boolean operators in English to be used in the search strategies are (Men who have sex with men OR gay) AND ((*Neisseria gonorrhoeae* OR *N. gonorrhoeae* OR Gonorrhoeae infection OR Gonorrhoeae) AND (*Chlamydia trachomatis* OR *C. trachomatis* OR *Chlamydia infection* OR Chlamydia)) AND (Africa OR Sub-Saharan Africa OR Western Africa OR Eastern Africa OR Southern Africa OR Central Africa). Also, a combination of relevant key words with names of each of the countries in Sub-Saharan Africa will also be performed. The search strategy will be adapted for each of the databases to be searched. The search strategy was piloted in April 2022 to test the appropriateness of selected keywords and electronic databases as depicted in Table 2.

**Table 2:** Potential search strategy on PubMed

Search	Query
#1	(“sexual and gender minorities”[MeSH Terms] OR men who have sex with men[Text Word]) OR (“sexual and gender minorities”[MeSH Terms] OR “homosexuality”[MeSH Terms] OR gay[Text Word])
#2	(((“neisseria gonorrhoeae”[MeSH Terms] OR Neisseria gonorrhoeae[Text Word]) OR (“neisseria gonorrhoeae”[MeSH Terms] OR N. gonorrhoeae[Text Word])) OR (gonorrhoeae[All Fields] AND (“infections”[MeSH Terms] OR infection[Text Word]))) OR gonorrhoeae[All Fields]
#3	(((“chlamydia trachomatis”[MeSH Terms] OR Chlamydia trachomatis[Text Word]) OR (“chlamydia trachomatis”[MeSH Terms] OR C. trachomatis[Text Word])) OR (“chlamydia infections”[MeSH Terms] OR chlamydia infection[Text Word])) OR (“chlamydia”[MeSH Terms] OR chlamydia[Text Word])
#4	(((((“africa”[MeSH Terms] OR Africa[Text Word]) OR (“africa south of the sahara”[MeSH Terms] OR Sub-Saharan Africa[Text Word])) OR (“africa, western”[MeSH Terms] OR Western Africa[Text Word])) OR (“africa, eastern”[MeSH Terms] OR Eastern Africa[Text Word])) OR (“africa, southern”[MeSH Terms] OR Southern Africa[Text Word])) OR (“africa, central”[MeSH Terms] OR Central Africa[Text Word])
#5	#1 AND #2 AND #3 AND #4

### Study selection

The selection of eligible studies will be based on the following inclusion and exclusion criteria:

#### Inclusion criteria

1. Studies that describe data from MSM in sub-Saharan Africa
2. Studies that include men who have sex with men 15 years and older
3. Studies that quantified the prevalence of chlamydia and/or gonorrhoea
4. Original research written in English language
5. Studies that report prevalence based on an adequate numerator and denominator where actual diagnostic tests were employed

#### Exclusion criteria

1. Studies that assessed non-human subjects
2. Studies published in languages other than English
3. Studies without full text available
4. Studies that computed incident infections only
5. Studies conducted in other countries other than countries in Sub-Saharan Africa
6. Case reports, Short reports, Letters, Notes, Conference abstracts, Review articles

**Fig. 1:**
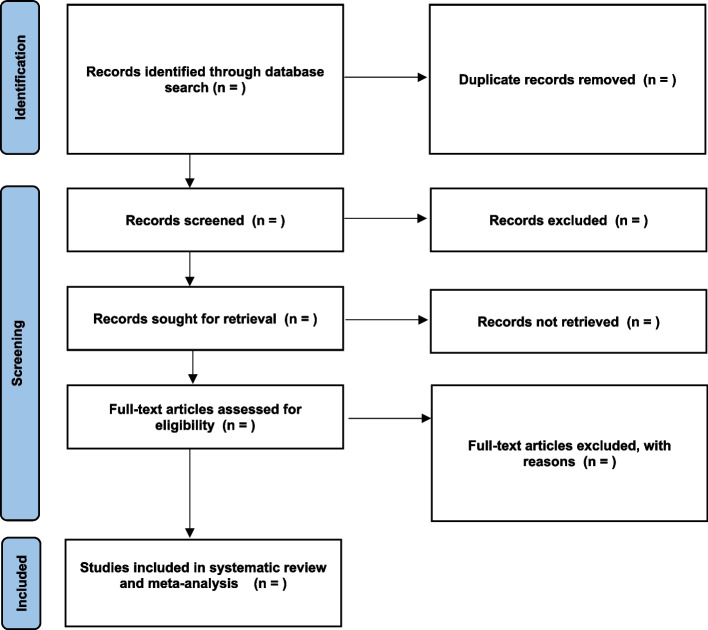
PRISMA flow diagram describing selection of studies for systematic review of gonorrhoea and chlamydia prevalence among MSM in sub-Saharan Africa [26]

First, title and abstracts of retrieved articles will be screened. Titles and abstracts of all articles identified from the search will be independently screened by the primary author and a co-author, following removal of duplicates. Then, full article screening will be conducted for their eligibility. Eligible articles will be retrieved and exported to Endnote version 20 reference manager. A hand search of the reference list of all selected articles will also be performed in order to be more comprehensive in the search strategy. Disagreements between the two authors (if any) will be discussed and resolved with the third and fourth authors. Full article screening based on eligibility criteria will then be conducted independently by two authors and all discrepancies resolved by the third and fourth authors. The PRISMA flowchart will be used to report the screening results (Fig. 1).

### Data extraction

A data extraction template will be designed using Microsoft Excel for collection of data from eligible studies. This data extraction template will be piloted and edited through an iterative process. Two authors will independently extract the data. The third and fourth author will independently verify all extracted data. The following data will be extracted: first author, year of publication, study period, country, geographic location, study design, setting, type of participants, sampling method, sample size, age, median age, specimen type, anatomical site, laboratory diagnosis method, prevalence of chlamydia and/or gonorrhoeae (Table 3). In case of missing or incomplete information, authors will contact the authors of the publications to request further particulars.

**Table 3:** Data extraction tool

1. First author
2. Year of publication
3. Study period
4. Country
5. Geographic location
6. Study design
7. Setting
8. Type of participants
9. Sampling method
10. Sample size
11. Age
12. Mean age
13. Specimen type
14. Anatomical site
15. Laboratory diagnosis method
16. Prevalence of chlamydia and/or gonorrhoeae

### Risk of bias and quality assessment

The risk of bias tool for prevalence studies will be used to evaluate the quality and risk of bias of the included studies for the review and meta-analysis. This tool which was developed by Hoy and colleagues uses a 10-item rating scale to assess the internal and external validity of studies [27]. Each of the 10 items will be rated as either low or high risk of bias and the overall risk of bias will then be determined according to the number of high risk of bias per study (low: ≤2; moderate: 3–4; and high: ≥5) [28]. Insufficient information related to 10 items will be regarded as high risk of bias [29, 30].

The quality of evidence provided by the included studies will be established using the Grading of Recommendations, Assessment, Development and Evaluation (GRADE) tool taking into account the risk of bias, indirectness of evidence, inconsistencies, imprecision and publication bias. Following assessment, the overall certainty in evidence will be categorized into four: High (the true effect is similar to the estimated effect), Moderate (where the true effect is probably close to the estimated effect), Low (where the true effect might be markedly different from the estimated effect) and Very low (where the true effect is probably markedly different from the estimated effect).

### Data synthesis and analysis

The extracted data will be analysed using the MetaXL add-on for Microsoft excel [31, 32]. The data will be presented using tables and the results will be graphically presented in forest plots. The heterogeneity in the studies will be evaluated using the *I*^*2*^ statistic. Where heterogeneity is high and significant across the included studies, a random effect model will be used to determine the pooled prevalence estimate. Subgroup analysis and meta-regression will be conducted to detect possible sources of heterogeneity according to study characteristics such as study design, sample size, country, diagnostic test and HIV status. Publication bias will be assessed using the Doi plot [33].

## Discussion

In this study, we expect to be able to systematically determine the prevalence of Ng and Ct among MSM. Furthermore, we will explore possible risk factors associated with prevalence. The results section of the systematic review and meta-analysis will include a description of all studies, results of all analyses including planned subgroup analyses. We will in the discussion section summarize the main findings and their implications. We will also compare our findings with others and discuss limitations of the study.

This study is necessary and of importance considering that STIs remain a major public health problem in Africa, and the role of MSM in the transmission dynamics is increasingly being recognized [34]. Published estimates indicate that Africa remains the continent most affected by STI/HIV [35]. MSM, particularly those from Africa, are at increased risk for STIs such as HIV, Ng, Ct and syphilis [34, 36, 37] and may experience significant barriers to quality health care due to widespread sigma, criminalization [38] and ridicule by healthcare workers [22]. This situation is compounded by the general lack of data on MSM prevalence of STI/HIV and risk factors in the African setting [34, 35]. Therefore, in order to adequately address and contain the STI epidemic and the associated burden in this key population, more research is required.

The outcomes of the systematic review and meta-analyses will be disseminated through publication in peer-reviewed journals. The findings of this study will serve to support researchers and public health stakeholders in identifying healthcare priorities and in addressing issues pertaining to the overall wellbeing of the MSM population.

## Data Availability

All data generated or analysed during this investigation will be included in the published systematic review article and will be available upon request.

## Abbreviations

STIs: Sexually transmitted infections
HIV: Human immunodeficiency virus
MSM: Men who have sex with men
Ng: *Neisseria gonorrhoeae*
Ct: *Chlamydia trachomatis*
PRISMA: Preferred Reporting Items for Systematic Reviews and Meta-analyses
PRISMA-P: Preferred Reporting Items for Systematic Reviews and Meta-analyses Protocols
PEO: Population Exposure Outcomes
DOAJ: Directory of Open Access Journals
MeSH: Medical Subject Headings
GRADE: Grading of Recommendations, Assessment, Development and Evaluation

## References

[1] Fleming DT, Wasserheit JN (1999). From epidemiological synergy to public health policy and practice: the contribution of other sexually transmitted diseases to sexual transmission of HIV infection. Sex Transm Infect.

[2] De Schryver A, Meheus A (1990). Epidemiology of sexually transmitted diseases: the global picture. Bull World Health Organ.

[3] Galvin SR, Cohen MS (2004). The role of sexually transmitted diseases in HIV transmission. Nat Rev Microbiol.

[4] World Health Organization (2012). Global incidence and prevalence of selected curable sexually transmitted infections.

[5] Newman L, Rowley J, Vander Hoorn S, Wijesooriya NS, Unemo M, Low N, Stevens G, Gottlieb S, Kiarie J, Temmerman M (2015). Global estimates of the prevalence and incidence of four curable sexually transmitted infections in 2012 based on systematic review and global reporting. PLoS One.

[6] World Health Organization (2018). Report on global sexually transmitted infection surveillance 2018.

[7] Wade AS, Kane CT, Diallo PA (2005). HIV infection and sexually transmitted infections among men who have sex with men in Senegal. AIDS.

[8] Vuylsteke B, Semde G, Sika L (2012). High prevalence of HIV and sexually transmitted infections among male sex workers in Abidjan, Cote d’Ivoire: need for services tailored to their needs. Sex Transm Infect.

[9] Quilter LAS, Obondi E, Kunzweiler C (2019). Prevalence and correlates of and a risk score to identify asymptomatic anorectal gonorrhoea and chlamydia infection among men who have sex with men in Kisumu Kenya. Sex Transm Infect.

[10] Ramadhani HO, Liu H, Nowak RG (2017). Sexual partner characteristics and incident rectal *Neisseria gonorrhoeae* and *Chlamydia trachomatis* infections among gay men and other men who have sex with men (MSM): a prospective cohort in Abuja and Lagos Nigeria. Sex Transm Infect.

[11] Buve A, Weiss HA, Laga M (2001). The epidemiology of gonorrhoea, chlamydial infection and syphilis in four African cities. AIDS.

[12] Ndiaye AG, Faye CM, Ndiaye I (2009). Screening for HIV, Syphilis, *Chlamydia trachomatis* and *Neisseria gonorrhoeae* during a combined survey conducted in Malicouna, a Senegalese rural area. Bull Soc Pathol Exot.

[13] Wade AS, Larmarange J, Diop AK (2010). Reduction in risk-taking behaviours among MSM in Senegal between 2004 and 2007 and prevalence of HIV and other STIs. ELIHoS Project, ANRS 12139. AIDS Care.

[14] Keshinro B, Crowell TA, Nowak RG (2016). High prevalence of HIV, chlamydia and gonorrhoea among men who have sex with men and transgender women attending trusted community centres in Abuja and Lagos, Nigeria. J Int AIDS Soc.

[15] Hessou PHS, Glele-Ahanhanzo Y, Adekpedjou R (2019). Comparison of the prevalence rates of HIV infection between men who have sex with men (MSM) and men in the general population in sub-Saharan Africa: a systematic review and meta-analysis. BMC Public Health.

[16] Baral S, Trapence G, Motimedi F (2009). HIV prevalence, risks for HIV infection, and human rights among men who have sex with men (MSM) in Malawi, Namibia, and Botswana. PLoS One.

[17] Graham SM, Mugo P, Gichuru E (2013). Adherence to antiretroviral therapy and clinical outcomes among young adults reporting high- risk sexual behaviour, including men who have sex with men, in coastal Kenya. AIDS Behav.

[18] Tafuma TA, Merrigan MB, Okui LA, Lebelonyane R, Bolebantswe J, Mine M, Chishala S, Moyo S, Thela T, Rajatashuvra A (2014). HIV/sexually transmitted infection prevalence and sexual behaviour of men who have sex with men in 3 districts of Botswana: results from the 2012 Biobehavioural Survey. Sex Transm Dis.

[19] Dudareva-Vizule S, Haar K, Sailer A (2014). Prevalence of pharyngeal and rectal *Chlamydia trachomatis* and *Neisseria gonorrhoeae* infections among men who have sex with men in Germany. Sex Transm Infect.

[20] Rebe K, Lewis D, Myer L (2015). A cross sectional analysis of gonococcal and chlamydial infections among men-who-have-sex-with-men in Cape Town, South Africa. PLoS One.

[21] Carroll A, Mendos LR. State sponsored homophobia 2017: a world survey of sexual orientation laws: criminalisation, protection and recognition. 12th ed. International Lesbian, Gay, Bisexual, Trans and Intersex Association (ILGA); 2017. https://www.ecoi.net/en/file/local/1399981/90_1495430692_ilga-state-sponsored-homophobia-2017-web-corr.pdf. Accessed 7 Apr 2022.

[22] Smith AD, Tapsoba P, Peshu N, Sanders EJ, Jaffe HW (2009). Men who have sex with men and HIV/AIDS in sub-Saharan Africa. Lancet.

[23] Poteat T, Diouf D, Drame FM (2011). HIV risk among MSM in Senegal: a qualitative rapid assessment of the impact of enforcing laws that criminalize same sex practices. PLoS One.

[24] Beyrer C, Baral SD, Van Griensven F (2012). Global epidemiology of HIV infection in men who have sex with men. Lancet.

[25] Shamseer L, Moher D, Clarke M (2015). Preferred reporting items for systematic review and meta-analysis protocols (PRISMA-P) 2015: elaboration and explanation. BMJ.

[26] Page MJ, McKenzie JE, Bossuyt PM, Boutron I, Hoffmann TC, Mulrow CD (2021). The PRISMA 2020 statement: an updated guideline for reporting systematic reviews. BMJ.

[27] Hoy D, Brooks P, Woolf A, Blyth F, March L, Bain C (2012). Assessing risk of bias in prevalence studies: modification of an existing tool and evidence of interrater agreement. J Clin Epidemiol.

[28] Tezera R, Sahile Z, Yilma D (2018). Prevalence of anaemia among school-age children in Ethiopia: a systematic review and meta-analysis. Syst Rev.

[29] Dorsamy V, Bagwandeen C, Moodley J (2022). The prevalence, risk factors and outcomes of anaemia in South African pregnant women: a systematic review and meta-analysis. Syst Rev.

[30] Young JJ, Hartvigsen J, Jensen RK (2020). Prevalence of multimorbid degenerative lumbar spinal stenosis with knee and/or hip osteoarthritis: protocol for a systematic review and meta-analysis. Syst Rev.

[31] Barendregt JJ, Doi SA, Lee YY, Norman RE, Vos T (2013). Meta-analysis of prevalence. J Epidemiol Community Health.

[32] Doi SA, Barendregt JJ, Khan S, Thalib L, Williams GM (2015). Simulation comparison of the quality effects and random effects methods of meta-analysis. Epidemiology.

[33] Furuya-Kanamori L, Barendregt JJ, Doi SAR (2018). A new improved graphical and quantitative method for detecting bias in meta-analysis. Int J Evid Based Healthc.

[34] Muraguri N, Tun W, Okal J, Broz D, Raymond HF, Kellogg T, Dadabhai S, Musyoki H, Sheehy M, Kuria D, Kaiser R, Geibel S (2015). HIV and STI prevalence and risk factors among male sex workers and other men who have sex with men in Nairobi Kenya. J Acquir Immune Defic Syndr..

[35] Lewis DA (2011). HIV/sexually transmitted infection epidemiology, management and control in the IUSTI Africa region: focus on sub-Saharan Africa. Sex Transm Infect.

[36] World Health Organization (2006). Report on global sexually transmitted infection surveillance 2015.

[37] Centers for Disease Control and Prevention (2017). Sexually transmitted disease surveillance 2016. United States Department of Health and Human Services..

[38] African Medical and Research Foundation (Amref Health Africa) (2017). Position statement on MSM.

